# The future of PrEP: novel long-acting HIV prevention agents for adolescent women

**DOI:** 10.11604/pamj.2024.47.102.36052

**Published:** 2024-03-04

**Authors:** Sharon Owuor, Makobu Kimani, Richard Kaplan

**Affiliations:** 1 Kenya Medical Research Institute, Wellcome Trust Research Programme, Kilifi, Kenya,; 2 The Desmond Tutu HIV Centre, Department of Medicine, Faculty of Health Sciences, University of Cape Town, Cape Town, South Africa

**Keywords:** PrEP, adolescent women, HIV

## Abstract

Adolescent girls and young women in Africa are at high risk of HIV and should be considered a key population for HIV prevention initiatives. Oral Tenofovir/Emtricitabine as pre-exposure prophylaxis (PrEP) has been shown to be effective on an individual and population level among key populations in Europe, Australia, and the US. However, studies in sub-Saharan Africa in a generalised epidemic have been less promising with adherence to daily tablets identified as a major problem. Long-acting PrEP drugs are being developed as a response to this problem. The first of these long-acting agents, injectable Cabotegravir given every two months has shown superiority to oral PreP and has been approved by the US Food and Drug Administration (FDA). Another long-acting PrEP drug in development is Lenacapavir which is an investigational, first-in-class long-acting HIV-1 capsid inhibitor that can be given as a six-monthly injection. These long-acting drugs could be a highly effective public health HIV prevention intervention. If made readily available to a vulnerable population of adolescent young women who are at high risk of HIV they could play an important role in protecting this key population against HIV and potentially reduce the population level risk of HIV.

## Introduction

In Africa, sexually active adolescent girls and young women (AGYW) are a vulnerable population at increased risk of HIV acquisition coupled with a largely unmet need for HIV prevention options. The highest numbers of HIV positive adolescents live in sub-Saharan Africa. As at 2020, there were 1.75 million adolescents living with HIV/AIDS worldwide, with 88% (1.5 million) of these adolescents living in sub-Saharan Africa [1]. Pre-exposure prophylaxis (PrEP), has been shown to be effective at preventing HIV if adhered to, and could potentially be a viable option in meeting the needs of young women [2]. PrEP uptake in young women is particularly challenging due to multiple factors, key among them being the daily pill burden. Recent clinical trials have confirmed the safety and superior efficacy of long acting two monthly Cabotegravir PrEP injections compared to daily oral PrEP. This drug as well as other long-acting drugs still in development could have a marked impact on the HIV epidemic if provided to most at risk populations including adolescent women in Africa. The overall objective of this paper is to encourage the speedy rollout of long-acting injectable PrEP among one of the most vulnerable populations, adolescent girls and young women in Africa.

## Commentary

**Key populations:** key populations are the people who are at the highest risk of contracting and transmitting HIV [3]. The Joint United Nations Programme on HIV/AIDS identifies five categories of key populations namely men who have sex with men (MSM), sex workers, prisoners, people who inject drugs and transgender people. In 2016, key populations and their sexual partners were reported to have accounted for 80% of new HIV infections outside sub-Saharan Africa and 25% of all new HIV infections in sub-Saharan Africa [4].

**Adolescent girls and young women as a key population:** while not meeting the traditional criteria for a key population, adolescent girls and young women in Africa have been identified as a population at high risk of HIV and therefore a potential population for focussed HIV prevention initiatives in the generalized HIV epidemic in Africa [5]. Key factors that predispose adolescent women to HIV are age-disparate sexual partnering and the notion of sponsors. A study of the HIV transmission patterns in KwaZulu-Natal reported that men aged 25-40 years were most likely to be transmitting the virus to women aged less than 25 years. These women then transmitted the virus to men in the same age bracket [5]. In Kenya, young people aged 15-24 years contributed 51% of the new HIV infections in 2015 and sponsors were shown to play a key role in this trend [6].

**HIV Incidence and Prevalence in Kenya:** Eastern and Southern Africa are the regions most affected by HIV in Africa. HIV prevalence is high in Kenya, with 1.5 million people living with HIV and 36 000 deaths occurring due to AIDS-related illnesses in 2015 [6]. In Eastern and Southern Africa, it is estimated that young women will acquire HIV 5-7 years earlier than young men [5]. Gender-disparities in HIV testing have also played a key role in this disproportionate distribution of HIV. Reports indicate that only 27% and 16% of adolescent girls and boys respectively have been tested.

**The efficacy of oral PrEP:** PrEP has been shown to be highly effective in reducing HIV incidence with studies indicating that PrEP is effective on an individual and community level for key populations [7]. PrEP studies among MSM in New South Wales, Australia and in Scotland have shown a decline in HIV incidence in the study populations as well as a reduction in the population-level risk of HIV in individuals who were not on PrEP when PrEP was implemented with routine care among key populations [7]. PrEP has also been shown to be moderately effective in preventing HIV infection in a heterosexual population in Botswana [8]. However, there are limited data to show that PrEP is effective on a population level in a generalized epidemic in sub-Saharan Africa. With adolescent women having been identified as a key population in a generalised epidemic, a targeted intervention focused on adolescent women in sub-Saharan Africa may be beneficial in reducing the pandemic both at an individual and societal level.

**PreP in Kenya:** Kenya´s PrEP programme is the largest in Africa, with approximately 25 000 people currently taking PrEP [9]. By rolling out PrEP as a national public sector programme, Kenya has rapidly scaled up PrEP programmes at HIV testing centres, key population drop-in centres, and maternal health clinics [10]. Despite these numerous efforts, the country faces the challenge of widespread PrEP discontinuation among users. The Prevention Options for Women Evaluation Research (POWER) study among young women was undertaken in Cape Town, Johannesburg, and Kisumu in Kenya to assess the persistence and patterns of TDF/FTC PrEP delivery among adolescent women. The study found that PrEP use was discontinued within 6 months in 80% of these women, while 20% of them persisted through 6 months. The adoption of flexible PrEP delivery patterns and long-acting injectable agents to enhance PrEP use among young women was recommended [11].

**Long-acting formulations:** the HIV Prevention Trials Network (HPTN) 083 and HPTN 084 clinical trials have confirmed the safety and efficacy of long acting cabotegravir (CAB LA) injections compared to daily oral PrEP. In the HPTN 083 study with transgender women and cisgender MSM, CAB LA as an injection given every eight weeks was shown to be superior to daily oral TDF/FTC [12]. The HPTN 084 study compared the safety and efficacy of CAB LA with daily oral TDF/FTC in HIV-uninfected cisgender women from sub-Saharan Africa aged 18-45 years. In this study, 3 224 study participants were designated to two study arms; ‘double dummy’ in which one arm received the eight-weekly active injectable CAB and inactive placebo oral TDF/FTC tablets whereas the second arm received the daily oral TDF/FTC and inactive injectable CAB. The study reported 40 breakthrough infections, with 36 infections in the women who were receiving oral TDF/FTC tablets and 4 infections in the women who were on active injectable CAB. Overall, the women in the CAB arm had 89% fewer infections than those in the daily oral TDF/FTC arm. CAB LA was safe and showed superior efficacy to daily oral TDF/FTC, making it a potential game-changer [13]. A mathematical modelling study that assessed the potential impact that CAB LA could have in preventing HIV in South Africa assessed the impact when the population groups were stratified as adults, men, women, high risk women and high-risk young women [14]. Overall, CAB LA was projected to avert 15% of new infections from 2023-2050 when delivered to 10% of the adult population. CAB LA effect was projected to be greatest in high-risk young women, a group that could benefit the most and have a knock-on effect on the entire population. In what could be a promising solution to the challenge of the pill burden, the US FDA has approved the first injectable CAB PrEP. It will be administered as two initial injections given one month apart, with the subsequent injections being administered every two-months [15]. A significant drawback of the drug is the cost with a market price of USD 3 700 per dose in the USA though no pricing has yet been announced for lower- and middle-income countries. The global health agency, Unitaid, has initiated a programme that will integrate CAB PrEP into national sexual health programmes to generate crucial real-world evidence that could underpin efforts for rapid global scale-up. The target groups for this programme will be cisgender men and transgender women in Brazil and adolescent girls and young women in South Africa [16]. These groups were selected based on their being disproportionately affected by HIV. This promising move is targeted at solving the two major challenges that oral PrEP has presented; the pill burden and stigma associated with taking pills. In a further recent development in July 2022, ViiV Healthcare granted a voluntary licence for long-acting injectable cabotegravir to the Medicines Patent Pool. This deal will allow low- and lower-middle income countries as well as all African countries regardless of income level to access affordable generic versions of Cabotegravir [17]. Another promising long-acting agent currently in development is Lenecapavir.

**Lenecapavir:** Lenacapavir (LEN) is an investigational, first-in-class long-acting capsid inhibitor which disrupts the HIV capsid at multiple stages throughout the viral life cycle [17]. Lenacapavir is being investigated both as therapy and for the prevention of HIV. LEN is also being investigated as a PrEP injection given every six months in the Purpose 1 trial in Adolescent Girls and Young Women and the Purpose 2 trial among MSM and Transgender people [18]. While the future development of this new long-working drug is still uncertain, it has the potential to provide new treatment and prevention options for HIV that could have a significant impact on the HIV epidemic. The recent outcomes of PrEP trials with Cabotegravir have shown its superiority to oral PrEP when dosed every two months as an intramuscular injection. If proven to be equally efficacious, Lenacapavir could also offer a long-acting alternative that could reduce the adherence problems that have caused oral PrEP to fail.

## Conclusion

These new long-acting drugs have the potential to provide more effective HIV prevention options than oral PrEP. If they are made readily available to a vulnerable population of adolescent young women who are at high risk of HIV, they could play an important role in protecting this key population against HIV and potentially reduce the population level risk of HIV, thereby significantly contributing towards alleviating the burden of HIV in Kenya and sub-Saharan Africa as a whole. With Cabotegravir already registered by the FDA in the US, the urgent registration and roll-out of this drug as PrEP for vulnerable populations in Africa is of crucial importance. The recent voluntary licensing of Cabotegravir with the Medicines Patent Pool could make this a highly cost effective public health intervention that should be supported by global health funders and public health policy makers in Africa.
